# Impact of Alpha-Ketoglutarate on Skeletal Muscle Health and Exercise Performance: A Narrative Review

**DOI:** 10.3390/nu16223968

**Published:** 2024-11-20

**Authors:** Miaomiao Xu, Qiao Zhang, Xiaoguang Liu, Liming Lu, Zhaowei Li

**Affiliations:** 1 School of Physical Education and Health, Guangzhou University of Chinese Medicine, Guangzhou 510405, China; miaomiaoxu@gzucm.edu.cn; 2 South China Research Center for Acupuncture and Moxibustion, Medical College of Acu-Moxi and Rehabilitation, Guangzhou University of Chinese Medicine, Guangzhou 510405, China; 3 College of Sports and Health, Guangzhou Sport University, Guangzhou 510500, China

**Keywords:** alpha-ketoglutarate, skeletal muscle regeneration, muscle atrophy, exercise performance

## Abstract

AKG, a central metabolite in the Krebs cycle, plays a vital role in cellular energy production and nitrogen metabolism. This review explores AKG’s potential therapeutic applications in skeletal muscle health and exercise performance, focusing on its mechanisms for promoting muscle regeneration and counteracting muscle atrophy. A literature search was conducted using the PubMed, Web of Science, and Scopus databases, yielding 945 articles published up to 31 October 2024. Of these, 112 peer-reviewed articles met the inclusion criteria and formed the basis of this review. AKG supports muscle recovery by stimulating muscle satellite cells (MuSCs) and macrophage polarization, aiding muscle repair and reducing fibrosis. Additionally, AKG shows promise in preventing muscle atrophy by enhancing protein synthesis, inhibiting degradation pathways, and modulating inflammatory responses, making it relevant in conditions like sarcopenia, cachexia, and injury recovery. For athletes and active individuals, AKG supplementation has enhanced endurance, reduced fatigue, and supported faster post-exercise recovery. Despite promising preliminary findings, research gaps remain in understanding AKG’s long-term effects, optimal dosage, and specific pathways, particularly across diverse populations. Further research, including large-scale clinical trials, is essential to clarify AKG’s role in muscle health and to optimize its application as a therapeutic agent for skeletal muscle diseases and an enhancer of physical performance. This review aims to provide a comprehensive overview of AKG’s benefits and identify future directions for research in both clinical and sports settings.

## 1. Introduction

**Figure 1:**
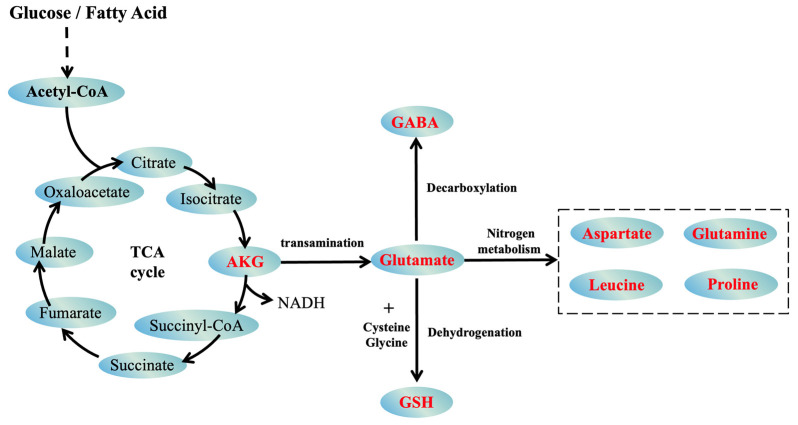
Metabolic role of AKG in the TCA cycle and nitrogen metabolism. This figure illustrates AKG’s role within the TCA cycle, in which it facilitates the conversion of isocitrate to succinyl-CoA, generating NADH for ATP synthesis. AKG also participates in nitrogen metabolism through transamination, producing glutamate, which serves as a precursor for amino acids like GABA, GSH, and essential amino acids, such as aspartate, glutamine, leucine, and proline. These pathways underscore AKG’s contribution to cellular energy production, amino acid synthesis, and nitrogen balance. TCA, tricarboxylic acid; AKG, alpha-ketoglutarate; NADH, nicotinamide adenine dinucleotide; GSH, glutathione; GABA, gamma-aminobutyric acid.

Alpha-ketoglutarate (AKG) is a pivotal metabolic intermediate within the Krebs cycle, also known as the tricarboxylic acid (TCA) cycle, that is essential for cellular energy production. In this metabolic pathway, AKG participates in the oxidative decarboxylation of isocitrate, facilitating its conversion into succinyl-CoA. This reaction releases carbon dioxide and generates NADH (Figure 1), which is a critical molecule that contributes to ATP synthesis through oxidative phosphorylation [1]. ATP subsequently powers many cellular processes, underscoring AKG’s role in sustaining cellular energy production. Additionally, AKG serves as a precursor in various biosynthetic pathways, including synthesizing amino acids such as glutamate and glutamine. These amino acids are critical for protein synthesis, neurotransmission, and detoxification, thereby supporting cellular repair and function [2,3].

In addition to its role in energy production, AKG is a key player in nitrogen metabolism, acting as a nitrogen acceptor in transamination reactions, where it helps balance nitrogen levels by converting amino acids into glutamate (Figure 1). This function is particularly important for muscle tissues, which are heavily involved in protein turnover and rely on AKG to ensure effective nitrogen utilization and detoxification through the urea cycle [4]. Its role in maintaining nitrogen balance underlines AKG’s broader contribution to metabolic homeostasis, ensuring that cells can function optimally, especially during periods of metabolic stress, such as muscle injury or intense physical activity [5].

Given its significant role in both energy and nitrogen metabolism, AKG has drawn increasing attention for its potential therapeutic applications in skeletal muscle health [6]. Skeletal muscle diseases and exercise performance are two critical research areas due to their substantial impact on health and quality of life [7]. Muscle wasting diseases, such as sarcopenia and cachexia [8,9,10], are prevalent among the aging population and individuals with chronic illnesses, contributing to physical frailty, reduced mobility, and increased mortality [11,12]. Despite advancements in understanding the pathophysiology of these diseases, effective treatments remain limited [13]. Metabolic modulators, like AKG, have emerged as potential interventions due to their role in energy production, protein metabolism, and oxidative stress reduction [14], all of which are implicated in the progression of muscle diseases [15]. Understanding how AKG modulates these processes could open new avenues for therapeutic approaches to mitigate muscle degeneration and improve patient outcomes [16,17].

Similarly, the interest in improving exercise performance has also led to the exploration of metabolic compounds like AKG [3]. AKG’s role in cellular energy production and protein synthesis has spurred research into its effects on exercise performance, particularly in enhancing endurance, reducing fatigue, and promoting muscle recovery [18,19]. Exercise places significant metabolic demands on the body, particularly in skeletal muscle, where efficient energy production and recovery are crucial for performance [20]. Athletes and physically active individuals seek ways to enhance these processes, with AKG being investigated for its potential to boost endurance, reduce fatigue, and support faster muscle recovery after exercise [21,22]. While initial studies have shown promising results, research gaps remain regarding the optimal dosing, long-term effects, and specific mechanisms through which AKG benefits exercise physiology.

Despite the growing interest in AKG’s role in skeletal muscle diseases and exercise performance [23,24], significant gaps in the research persist. Much of the current evidence comes from preclinical studies or small-scale clinical trials, and the underlying mechanisms by which AKG exerts its effects are not fully understood [25]. Additionally, there is a need for more rigorous clinical trials to determine the efficacy and safety of AKG supplementation, especially in diverse populations, including senior citizens, individuals with muscle disorders, and athletes [24,26,27] (Table 1). The interactions between AKG and other metabolic pathways, its potential synergistic effects with other nutrients, and its long-term impact on muscle health and performance require further investigation [28,29].

This review aimed to critically examine the role and mechanisms of AKG in skeletal muscle function, focusing on its potential applications in muscle disease treatment and exercise performance enhancement. By exploring the current literature on AKG’s metabolic functions, particularly its effects on muscle protein turnover, inflammation, and energy production, this review aims to provide a comprehensive overview of the therapeutic potential of AKG in these contexts. Additionally, this review will highlight key areas in which further research is needed to fully understand AKG’s role and optimize its use in both clinical and sports settings.

## 2. Methods

This narrative review conducted a comprehensive literature search using the PubMed, Web of Science, and Scopus databases to identify studies on the potential applications of AKG in skeletal muscle health and exercise performance. The research strategy included the following search terms: “Alpha-Ketoglutarate”, “Skeletal Muscle Health”, and “Exercise Performance”. Additional keywords, such as “Muscle Regeneration”, “Muscle Atrophy”, “Protein Synthesis”, and “Inflammation”, were utilized when relevant to capture the diverse mechanisms of AKG. The search was limited to peer-reviewed articles published up to 31 October 2024. The initial search yielded 945 articles. To ensure the inclusion of high-quality and reliable studies, we assessed the quality of each study using the GRADE system, classifying them as high, moderate, or low quality. Studies with a high risk of bias were excluded to prevent any disproportionate influence on the results. After screening for relevance, studies examining the effects of AKG on muscle regeneration, muscle atrophy prevention, and inflammation modulation in both animal and human models were included. Papers not aligned with the objectives of the review were excluded, resulting in a total of 112 articles that met the inclusion criteria and formed the foundation of this review.

In our literature review, we did not find any systematic reviews on AKG supplementation that addressed the same topics as our review. Additionally, we confirmed that no similar review on AKG supplementation is currently registered. We observed that existing reviews on AKG focus primarily on general metabolic functions rather than on specific applications in skeletal muscle health and exercise performance.

## 3. AKG and Skeletal Muscle Health

AKG is a critical intermediate metabolite in the TCA cycle and regulates skeletal muscle health. In this section, we will discuss the role of AKG in skeletal muscle regeneration and atrophy. Most of the studies in this section are based on animal research.

### 3.1. AKG and Skeletal Muscle Regeneration

Skeletal muscle, constituting a significant proportion of human mass—exceeding 40%—fulfills pivotal roles in locomotion, postural maintenance, metabolic regulation, and respiratory function [30,39,40]. Skeletal muscle injuries are common occurrences in daily life and sports, and their regeneration involves a complex process with multiple stages and biological mechanisms [38,41,42]. According to our previous studies, skeletal muscle injury repair typically goes through three stages: the acute inflammatory and degenerative phase, the repair phase, and the remodeling phase [43,44,45,46]. Muscle satellite cells (MuSCs) are essential for maintaining skeletal muscle homeostasis and play a critical role in muscle regeneration following injury [47,48,49].

The number of MuSCs decreases with aging [50,51], accompanied by a decline in AKG levels. Additionally, AKG levels are significantly lower in primary myoblasts from aging mice compared to adult mice [52]. Supplementation with AKG increased the proliferation of aging mouse myoblasts. Consistent with these results, the injection of AKG promoted the proliferation of cardiomyocytes during heart development and after myocardial infarction [34]. Can AKG improve skeletal muscle regeneration in aging mice after skeletal muscle injury? Ciuffoli et al. (2024) found that intraperitoneal injections of AKG administered before injury and on days 1, 3, and 5 post-injury significantly increased the number of MuSCs and the cross-sectional area of regenerating skeletal muscle fibers compared to vehicle-treated mice [52]. Moreover, supplementation with AKG decreased fibrosis in injured skeletal muscle 28 days after CTX injection [52]. These results suggest that AKG may act as a supplement for treating skeletal muscle injury in aging mice by promoting MuSC proliferation.

Macrophages represent another crucial cellular component in the process of skeletal muscle regeneration following injury [53,54]. In our previous study, we found that macrophage deletion impaired skeletal muscle regeneration in a contused skeletal muscle mouse model [43]. AKG influences macrophage polarization, shifting the balance from pro-inflammatory M1 macrophages to anti-inflammatory M2 macrophages in bone healing and regeneration [36]. We speculate that AKG promotes the transition of macrophages from the M1 to the M2 phenotype during muscle regeneration after injury. However, further studies are needed to explore the role and mechanism of AKG in macrophage polarization during skeletal muscle regeneration.

**Figure 2:**
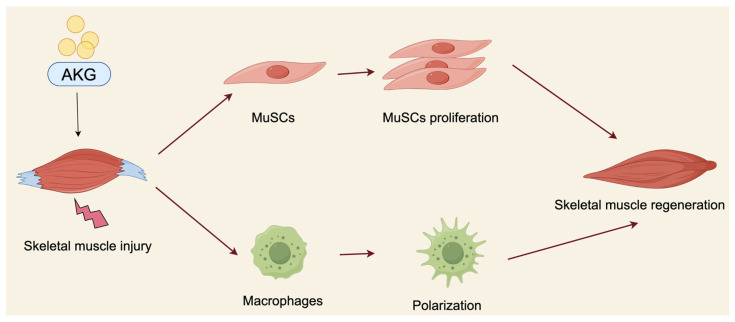
Role of AKG in skeletal muscle regeneration. The diagram illustrates how AKG contributes to skeletal muscle regeneration by promoting MuSCs’ proliferation and macrophage polarization. AKG, alpha-ketoglutarate; MuSCs, muscle satellite cells.

Taken together, AKG is a promising target for treating skeletal muscle injury by regulating MuSC proliferation and macrophage polarization (Figure 2).

### 3.2. AKG and Skeletal Muscle Atrophy

We and other groups have found that various pathological conditions, such as chronic illnesses, neoplastic processes, persistent infections, and aging, can disrupt the equilibrium between skeletal muscle protein synthesis and degradation, thereby initiating muscle wasting and potentially leading to skeletal muscle atrophy [55,56,57]. Skeletal muscle atrophy is characterized by a diminished capacity for force generation, increased susceptibility to fatigue, and reduced exercise tolerance, all collectively contributing to a diminished quality of life [58,59,60]. Skeletal muscle atrophy is always associated with metabolic remodeling, and various metabolites can serve as therapeutic targets for skeletal muscle atrophy [61,62]. For example, musclin, which is released from skeletal muscle after exercise, inhibits fibro-adipogenic progenitor (FAP) proliferation and promotes apoptosis, thereby protecting against skeletal muscle atrophy in a model of muscle disuse [63].

AKG is an important endogenous metabolite that is also involved in regulating skeletal muscle atrophy. Corticosterone is central to the hypothalamic–pituitary–adrenal (HPA) axis’s function, playing a key role in the body’s stress response and maintenance of various physiological processes [64]. It can also induce protein degradation and muscle atrophy. However, AKG effectively mitigates corticosterone-induced skeletal muscle protein degradation and muscle atrophy both in vivo in mice and in vitro in C2C12 myotubes [5]. In addition, Li et al. (2023) demonstrated that AKG supplementation significantly promotes the formation of myotubes, enhances energy metabolism, and reduces reactive oxygen species [65].

An imbalance between protein synthesis and degradation is a common feature of various types of skeletal muscle atrophy [33,66,67,68]. The mTOR signaling pathway is crucial for protein synthesis, with high activation associated with skeletal muscle hypertrophy and inhibition associated with decreased protein synthesis and skeletal muscle atrophy [31,69]. AKG supplementation increased mTOR, S6 kinase beta 1 (S6K1), and S6 phosphorylation in osteoblast cells [37]. Consistent with this result, supplementing the diet of lipopolysaccharide-challenged piglets with AKG activated the mTOR signaling pathway and improved skeletal muscle energy status [70]. AKG supplementation in a low-protein diet significantly improved the growth performance of piglets and activated the mTOR signaling pathway in skeletal muscle [6]. Additionally, AKG supplementation increases the availability of free amino acids in serum and muscle tissues and upregulates the expression of amino acid transporters in skeletal muscle, thereby activating the mTOR pathway and promoting muscle protein synthesis [71]. All these studies indicate that AKG inhibits muscle atrophy by enhancing protein synthesis pathways.

Major surgery is associated with losing skeletal muscle mass and function [72]. Might AKG mitigate surgery-induced skeletal muscle atrophy? In a study by Wernerman et al. (1987), supplementing total parenteral nutrition with ornithine-alpha-ketoglutarate (OKG) after cholecystectomy maintained skeletal muscle protein synthesis, as evidenced by stable ribosome concentration and polyribosome percentage, unlike the control group, which showed a decrease [73]. Additionally, OKG supplementation resulted in significantly lower urinary urea excretion, indicating improved nitrogen retention and utilization [73]. The study suggests that OKG could enhance nutritional support after major surgery by preserving muscle protein synthesis and nitrogen balance.

Duchenne muscular dystrophy (DMD) is a severe muscle atrophy disease caused by mutations in the dystrophin gene [72,74]. Although AKG effectively mitigates corticosterone- and surgery-associated skeletal muscle atrophy, whether it can inhibit DMD progression still requires further study. Mdx mice are widely used to study the mechanisms of DMD [75,76]. Cai et al. (2018) found that AKG supplementation (2% body weight) in drinking water effectively improved limb skeletal muscle strength, endurance, body weight, gastrocnemius weight, and soleus muscle weight in mdx mice compared with the vehicle group [5]. In vivo and in vitro results showed that the proline hydroxylase 3 (PHD3)/β2 adrenergic receptor (ADRB2) pathway is the key signaling mechanism, by which AKG protects against skeletal muscle atrophy [5]. PHD3 is a key regulator involved in various skeletal muscle pathophysiology, including skeletal muscle atrophy [77,78]. Decreasing PHD3 levels in vivo and in vitro can alleviate skeletal muscle atrophy by inhibiting the NF-κB signaling pathway [79]. ADRB2, as a target of PHD3, is also involved in regulating skeletal muscle atrophy. A study found that ADRB2 agonist clenbuterol attenuated ROS generation and skeletal muscle atrophy in uremic mice and C2C12 myotubes [80]. These results suggest that AKG effectively rescues skeletal muscle atrophy through the PHD3/ADRB2 pathway, which may serve as a potential treatment strategy for skeletal muscle atrophy in the future.

Inflammation and sepsis cause severe skeletal muscle atrophy, which, in turn, can increase the risk of morbidity and mortality [35,81,82]. The LPS-induced skeletal muscle atrophy mouse model is widely used to study inflammation- and sepsis-induced muscle atrophy [83,84,85]. Lei Wang et al. (2016) demonstrated that supplementing the diet of lipopolysaccharide-challenged piglets with AKG mitigated the loss of body weight after LPS treatment [70]. In this LPS model, inflammation was the primary cause of skeletal muscle atrophy [32,79,86]. AKG supplementation inhibits the inflammatory response by normalizing plasma concentrations of tumor necrosis factor α (TNF-α) and insulin-like growth factor 1 (IGF-1), both of which are disrupted by LPS challenge [70]. In skeletal muscle cells, TNF-α triggers the activation of the IκB kinase complex (IKK), leading to the phosphorylation and ubiquitination of IκB. This leads to the nuclear translocation of NF-κB and the expression of target genes, including the muscle-specific ubiquitin ligase MuRF1, involved in muscle protein degradation [87]. IGF-1 is a key growth factor that regulates skeletal muscle atrophy and hypertrophy through the PI3K/Akt/mTOR and PI3K/Akt/GSK3β pathways [88,89,90].

Lipotoxicity is a condition that occurs when excessive lipid accumulation in non-adipose tissues [91,92], such as skeletal muscle, leads to cellular dysfunction, damage, and skeletal muscle atrophy [93,94]. AKG inhibits various types of skeletal muscle atrophy. Can it also alleviate lipotoxicity-related muscle atrophy? AKG supplementation in a high-fat, high-fructose diet (HFFD) (as sodium AKG in drinking water) appeared to alleviate increased glycolytic enzyme activities, TAG accumulation, glycogen depletion, and reduced protein levels in HFFD-fed mice [14]. TAG accumulation and glycogen depletion contribute to lipotoxicity [95,96]. AKG supplementation protects against these effects, suggesting a role in mitigating lipotoxicity-related skeletal muscle atrophy. However, further studies are needed to explore how AKG protects against lipotoxicity-related skeletal muscle atrophy.

**Figure 3:**
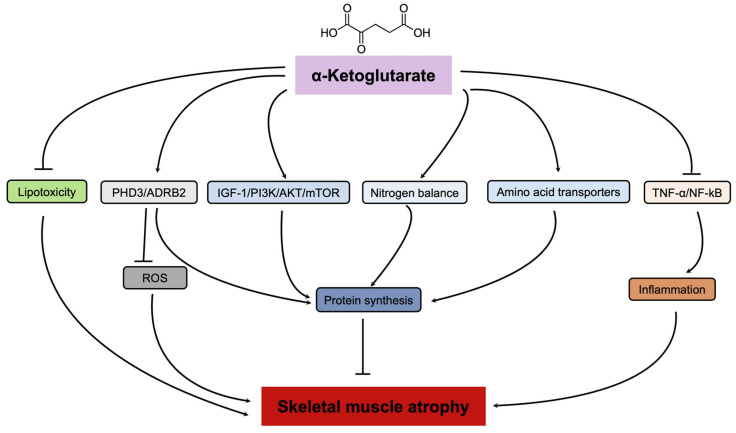
AKG’s role in alleviating skeletal muscle atrophy. The diagram illustrates how AKG alleviates skeletal muscle atrophy by inhibiting lipotoxicity, inflammation, ROS generation, and promoting protein synthesis. PHD3, proline hydroxylase 3; ADRB2, β2 adrenergic receptor; TNF-α, tumor necrosis factor α; IGF-1, insulin-like growth factor 1.

Overall, AKG supplementation could be a valuable strategy to protect against skeletal muscle atrophy by modulating protein degradation, protein synthesis, inflammation, and lipotoxicity (Figure 3).

## 4. AKG and Exercise Performance

Most of the studies in this section are based on human research. AKG, as a key intermediate metabolite in the TCA cycle, not only contributes to skeletal muscle regeneration and inhibits muscle atrophy but is also associated with improved exercise performance. L-arginine α-ketoglutarate (AAKG) is a compound composed of L-arginine and AKG, combining arginine’s blood flow improvement effects with the importance of AKG in energy metabolism [97]. Supplementing with AAKG can increase the levels of AKG in the body, promote energy production, and enhance amino acid utilization, thereby supporting basal metabolism and improving exercise performance [98,99,100]. Campbell et al. (2006) [97] found that AAKG significantly improved one repetition maximum (1RM) bench press performance compared to the placebo group in healthy adult men. Additionally, AAKG increased Wingate peak power, indicating improved anaerobic capacity. However, no significant differences were observed in body composition, total body water, isokinetic quadriceps muscle endurance, or aerobic capacity between the AAKG and placebo groups. In addition, AAKG was well-tolerated, with no significant changes in clinical blood markers, suggesting it is safe for use [97]. Furthermore, AKG supplementation improved muscle properties, such as increased shear force and drip loss, in laying hens [19]. For untrained men, AKG supplementation can increase training volume, maximum power output, and muscle torque, improve stress-recovery state, and enhance exercise tolerance and training effects [101]. Consistent with these results, 5-HMF/AKG supplementation briefly enhances exercise performance by improving maximal and lactate threshold running speeds after intensified training in soccer players (age: 14.7 ± 0.4 years) [102]. However, another study found no significant differences in maximal power output (powermax) during a short-term maximal exercise test between groups receiving pyridoxine-alpha-ketoglutarate (PAK) (50 mg/kg body mass), NaHCO_3_, both, or a placebo [103]. This suggests that PAK, in the dosage used, did not enhance short-term maximal exercise capacity in these well-trained cyclists. In summary, the studies indicate that AAKG supplementation can enhance exercise performance and muscle properties, with potential benefits for both trained and untrained individuals, while also being well-tolerated and safe for use.

Acute normobaric hypoxia is associated with poor exercise performance. Could AKG help mitigate this impairment? Mariacher et al. (2014) [104] found that 3 weeks of AKG (2.4 g/day) and 5-hydroxymethylfurfural (5-HMF) (90 mg/day) supplementation could prevent the decrease in aerobic exercise performance in acute normobaric hypoxia compared to the placebo group. However, another study found that supplementation with AKG (4.8 g/day) and 5-HMF (60 mg/day) did not prevent hypoxia-induced decreases in exercise performance [105]. All groups experienced a similar decline in maximal oxygen uptake (VO_2_max) and maximal power output under hypoxic conditions [105]. What might explain the differing results between these studies? We hypothesize that the more extended supplementation period in the first study might have allowed for more significant physiological adaptations, resulting in observed improvements in exercise performance under hypoxic conditions. In addition, although there were no significant improvements in exercise performance, there were still positive effects on muscle oxygen extraction. The more detailed mechanisms of these effects still need further exploration.

**Figure 4:**
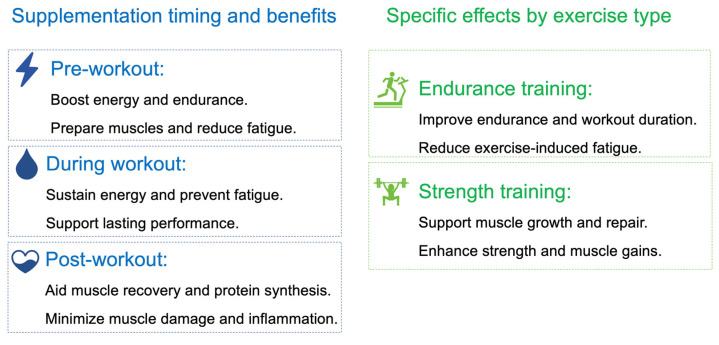
Timing and effects of AKG supplementation on exercise performance. Pre-workout AKG boosts energy and reduces fatigue; intra-workout sustains energy and performance; post-workout aids recovery and protein synthesis. AKG enhances endurance and reduces fatigue in endurance training while supporting muscle growth and strength in strength training.

Fatigue is a critical factor affecting exercise performance. Co-supplementation with calcium salt of β-hydroxy-β-methylbutyrate and L-arginine α-ketoglutarate significantly prevents decreases in countermovement jump height in young track and field athletes aged 14–17 years. Additionally, no significant differences in muscle damage markers were observed [100]. Overall, AKG has been shown to improve anaerobic performance, including bench press strength and Wingate peak power, without affecting body composition or aerobic capacity, and it is well-tolerated. Studies show mixed results on AKG’s effectiveness in mitigating hypoxia-induced performance declines, possibly due to differences in supplementation duration. Co-supplementation with other compounds may, however, help reduce fatigue in young athletes (Figure 4, Table 2).

## 5. Conclusions and Suggestion

In summary, AKG is a crucial intermediate metabolite in the TCA cycle and plays a significant role in preserving skeletal muscle health. AKG is not only involved in energy metabolism but also plays a crucial role in regulating muscle protein synthesis and degradation, oxidative stress, and nitrogen metabolism. Therefore, AKG supplementation shows considerable potential in improving skeletal muscle regeneration, preventing muscle atrophy, and enhancing exercise performance.

In skeletal muscle regeneration, AKG supports the repair process following muscle injury by promoting the proliferation of MuSCs and regulating macrophage polarization. AKG supplementation has demonstrated positive effects in treating age-related muscle damage. Additionally, AKG effectively mitigates muscle atrophy induced by chronic diseases, surgeries, and inflammation by regulating protein synthesis pathways, such as the mTOR pathway.

In exercise performance, AKG supplementation has shown promise in improving strength and endurance and reducing fatigue, particularly in high-intensity exercise or hypoxic environments. However, research indicates that different dosing regimens and durations of AKG supplementation may result in varying outcomes. Therefore, further research should optimize AKG dosage and administration timing to maximize its potential in exercise physiology.

Despite the promising applications of AKG in skeletal muscle diseases and exercise performance, current research primarily focuses on preclinical studies and small-scale clinical trials. More large-scale clinical trials are needed to confirm its long-term safety and efficacy. Additionally, understanding the interactions between AKG and other metabolic pathways and its synergistic effects with other nutrients is an important direction for future research.

## 6. Recommendations for AKG Supplementation in Human Populations

Current research suggests that AKG supplementation may benefit different human populations, including athletes, older adults, and individuals with specific health conditions. Recommendations for dosage, frequency, form of administration, and duration of supplementation are outlined below based on available evidence.

For athletes, studies indicate that daily doses of 3–4 g of AKG, administered 1–2 times per day, may improve endurance and anaerobic capacity. Supplementation for a period of 4–8 weeks appears to be effective, with administration typically in powder or capsule form. For immediate performance benefits, taking AKG before exercise is commonly recommended.

In the aging population, AKG supplementation at doses of approximately 2–3 g per day has been associated with muscle preservation and metabolic support. Regular intake for at least 8–12 weeks is suggested to achieve noticeable effects. Capsules are often preferred for convenience and tolerance in this group.

For individuals with specific health conditions, such as those experiencing muscle atrophy, AKG dosages of 2–4 g per day may help reduce muscle wasting and support recovery. However, in these cases, the duration and frequency of supplementation should be personalized and ideally guided by clinical recommendations, with particular consideration for potential drug or condition-specific interactions.

These recommendations serve as a preliminary guideline, but individual needs and health profiles should inform any specific supplementation regimen. Further research is warranted to refine optimal dosages and administration protocols across different populations and health contexts.

## Figures and Tables

**Table 1:** Effects of AKG on different organs and systems.

Organ/System	AKG Benefits	References
Muscle	AKG reduces muscle protein degradation and increases muscle strength and endurance.	[5,12,30]
Intestine	AKG helps maintain intestinal integrity and reduces damage to intestinal epithelial cells.	[20,31,32]
Liver	AKG lowers ammonia levels in the body and maintains the balance of nitrogen and proteins.	[14,21,30]
Kidney	AKG regulates the acid-base balance in renal tubules and enhances kidney function.	[16,31,33]
Cardiovascular	It helps prevent myocardial infarction, lowers blood pressure, and reduces cholesterol and triglyceride levels.	[15,23,34]
Brain	AKG reduces oxidative damage to neurons.	[19,29,35]
Bone	AKG reduces calcium loss in bones and minimizes the loss of minerals.	[12,36,37]
Skin	AKG promotes rapid wound healing and increases collagen production.	[19,26]
Cancer	AKG can inhibit the growth of cancer cells.	[8,9,28]
Immune System	AKG may influence immune function by regulating the metabolism of immune cells.	[2,28,38]

This table summarizes the multifunctional roles of AKG, highlighting its benefits across multiple organs and systems, including the muscles, intestines, liver, kidneys, cardiovascular system, brain, bones, skin, cancer, and immune system. AKG supports tissue integrity, regulates metabolic balance, provides cellular protection, and has potential therapeutic applications.

**Table 2:** Effects of AKG on exercise performance.

Population	AKG Treatment	Key Findings	References
35 resistance-trained men (age: 38.9 ± 5.8 years; height: 178 ± 8.4 cm; weight: 86 ± 13.7 kg).	4 g of AAKG was taken three times daily (12 g/day in total) for 8 weeks.	AKG reduces muscle protein degradation and increases muscle strength and endurance.	[97]
34 volunteers (Men: 14 (age: 15.1 ± 1.2 years; height: 171.4 ± 6.6 cm; weight: 64.6 ± 7.8 kg); Women: 20 (age: 15.0 ± 1.2 years; height: 158.9 ± 5.8 cm; weight: 52.6 ± 5.5 kg)).	Supplementation with 7.5 g of β-hydroxy-β-methylbutyrate and 10 g of AAKG daily for 12 days.	Daily co-supplementation of β-hydroxy-β-methylbutyrate calcium salt and AAKG may help prevent a decline in jump performance during training.	[100]
Untrained 33 healthy adult men (age: 26.7 ± 4.8 years; height: 178.3 ± 8.1 cm; weight: 81.6 ± 12.7 kg)	Daily supplementation of AKG at 0.2 g/kg/day for 4 weeks of training and 1 week of recovery.	AKG supplementation can increase training volume, maximum power output, and muscle torque, improve stress-recovery state, and enhance exercise tolerance and training effects.	[101]
17 youth soccer players (age: 14.7 ± 0.4 years)	Supplemented with 2 g AKG and 0.2 g 5-HMF in 200 mL water daily for 9 days during an in-season competition break.	5-HMF/AKG supplementation may briefly enhance exercise performance by improving maximal and lactate threshold running speeds after intensified training.	[102]
Healthy, trained males (age: 24.2 ± 2.6 years; height: 180.1 ± 5.2 cm; weight: 76.1 ± 7.4 kg).	2.4 g of AKG and 0.09 g 5-HMF per day (diluted in water, twice daily) for 21 days before the hypoxia test.	The decline of peak aerobic exercise performance in acute hypoxia was partly prevented by AKG and HMF.	[104]
Well-trained males (age: 25 ± 3 years; height: 179 ± 6 cm; weight: 76.4 ± 6.8 kg).	4.8 g of AKG and 60 mg of 5-HMF per day (in the form of liquid and capsules, twice daily) for 2 days before the hypoxia test.	AKG and 5-HMF did not prevent the performance decreases associated with acute hypoxia, but enhanced oxygen extraction in working muscles.	[105]
Medium distance runners, including 7 men (age: 15.0 ± 1 years; height: 173.0 ± 7.3 cm; weight: 66.0 ± 3.9 kg) and 12 women (age: 15.3 ± 1.3 years; height: 156.1 ± 7.2 cm; weight: 50.6 ± 3.8 kg).	1250 mg of calcium salt of β-hydroxy-β-methylbutyrate and 1250 mg of AAKG (capsule, three times a day) for 12 days.	β-hydroxy-β-methylbutyrate and AAKG prevent a decline in jump performance	[100]

AKG: alpha-ketoglutarate; AAKG: L-arginine alpha-ketoglutarate; 5-HMF: 5-hydroxymethylfurfural.

## Data Availability

The original contributions presented in the study are included in the article, further inquiries can be directed to the corresponding authors.
